# Matched-pair analysis of the impact of low-dose postoperative radiotherapy on prognosis in patients with advanced hypopharyngeal squamous cell carcinoma without positive surgical margins and extracapsular extension

**DOI:** 10.3389/fonc.2023.1089275

**Published:** 2023-09-07

**Authors:** Hengmin Tao, Yumei Wei, Zhong Shen, Zhichao Liu

**Affiliations:** Department of Head and Neck Radiotherapy, Shandong Provincial ENT Hospital, Shandong University, Jinan, China

**Keywords:** head and neck cancer, hypopharyngeal squamous cell carcinoma, postoperative radiotherapy, positive surgical margins, extracapsular extension

## Abstract

**Background:**

We conducted a comparative analysis between low and high-dose postoperative radiotherapy in patients with hypopharyngeal squamous cell carcinoma (HPSCC) in stage III or IV without positive surgical margins and extracapsular extension (ECE). Propensity score matching (PSM) was used to eliminate confounding factors and reduce bias.

**Methods:**

The matched-pair analysis included 156 patients divided into two groups: the low-dose radiotherapy group (LD-RT 50 Gy, 78 cases) and the high-dose radiotherapy group (HD-RT 60 Gy, 78 cases). Both cohorts were statistically comparable in terms of age, gender, subsite, and TNM classification.

**Results:**

The median follow-up time was 49 months (ranging from 5 to 100 months). The overall survival (OS) rate, progression-free survival (PFS) rate, locoregional control rate (87% vs. 85.7%; *p* = 0.754), distant metastases-free survival (79.2% vs. 76.6%; *p* = 0.506), and the occurrence of second primary tumors (96.1% vs. 93.5%; *p* = 0.347) showed no significant differences between the LD-RT group and the HD-RT group. The 3-year OS was 64.9% and 61% in the low-dose and high-dose group, respectively, and 63% in the entire group (p = 0.547). The 3-year PFS was 63.6% and 54.5% (*p* = 0.250), respectively, and the 3-year PFS of the entire group was 59.1%. Multivariate analyses revealed that pathological T and N classification, and pathological differentiation were associated with 3-year OS, PFS, and LRFS and were independent prognostic factors (*p* < 0.05). LD-RT was not associated with an increased risk of death and disease progression compared to HD-RT.

**Conclusion:**

The results of postoperative low-dose radiotherapy did not show inferiority to those of high-dose radiation for patients with advanced hypopharyngeal cancer without positive surgical margins and ECE in terms of OS, PFS, locoregional control, and metastases-free survival.

## 1 Introduction

It is well known that comprehensive therapy is essential for improving treatment outcomes and survival in patients with head and neck squamous cell carcinoma (HNSCC). Previous studies have shown that postoperative radiotherapy can improve local control and reduce recurrence. However, recommendations for adjuvant radiation doses have been inconsistent since the introduction of postoperative radiation in the 1950s by Fletcher, who suggested that 50 Gy was sufficient to control subclinical disease (1). In the 1990s, Peters et al. (2) proposed a guideline recommending 57.6 Gy to the surgical bed was adequate. In 2004, the EORTC and RTOG 95-01 recommended an adjuvant radiation dose range based on high-risk status (3, 4). The National Comprehensive Cancer Network (NCCN) published guidelines allowing a radiation dose of 66 Gy in 33 fractions for high-risk areas and 50 Gy in 25 fractions for low- to intermediate-risk areas (5). However, domestic guidelines recommend a postoperative adjuvant radiotherapy (PORT) dose of 60–66 Gy without specifying the fractionated dose. Despite the use of matched-pair analysis in several retrospective studies of head and neck cancer (6–9), LD-RT has not been compared with HD-RT in hypopharyngeal cancer in clinical practice, and there are no randomized studies comparing LD-RT with HD-RT. Therefore, to evaluate the efficacy of LD-RT in patients with locally advanced HPSCC without positive surgical margins and ECE, we conducted a matched-pair analysis to compare these two treatments in terms of overall survival (OS), progression-free survival (PFS), and locoregional control, based on our preliminary research (10).

## 2 Materials and methods

### 2.1 Patients

A total of 269 patients with HPSCC who received treatment at the Department of Head and Neck Surgery of our hospital between December 2013 and May 2019 were included in this retrospective study, all of whom underwent surgical treatment and postoperative radiotherapy. Among them, 269 patients were divided into two groups: the LD-RT group (50 Gy, 186 cases) and the HD-RT group (60 Gy, 83 cases). To reduce the impact of confounding factors and enhance homogeneity between the two groups, we employed propensity score matching (PSM). Ultimately, 78 patients treated with HD-RT were matched with 78 patients treated with LD-RT. The distribution of patient characteristics in both groups is presented in **Table 1**. Given the retrospective and observational nature of the study, informed consent was not required for inclusion. The mean age of the patients was 59 years (range, 37–81 years), and male patients constituted the majority (96%). Apart from the matched variables (T-classification, N-classification, and Interval surgery–radiotherapy), there were no statistically significant differences between the two groups in terms of gender, age, and degree of tumor differentiation (*p* > 0.05). Tumor staging was based on the 2010 American Joint Committee on Cancer (AJCC) TNM staging system (7th edition). High-risk factors were defined as pathological positive margins and ECE, while low-risk factors were defined as pathological T3 and T4 (without positive margins) and N1–3 (without ECE).

**Table 1:** Patient characteristics.

Characteristics	Patients	Low dose (50 Gy)	High-dose (>50 Gy)	Chi-square	*p*-value
Gender				0.693	0.405
Male	150	76	74		
Female	6	2	4		
Age				0.104	0.747
≤60 years	88	43	45		
>60 years	68	35	33		
Subsite				0.889	0.641
Pyriform sinus	118	60	58		
Posterior wall	27	14	13		
Postcricoid region	11	4	7		
Histological grade				0.176	0.916
Highly differentiated	6	3	3		
Moderately differentiated	122	60	62		
Poorly differentiated	28	15	13		
T-stage					Matched
1	0	0	0		
2	46	23	23		
3	72	36	36		
4	38	19	19		
N-stage					Matched
0	12	6	6		
1	18	9	9		
2	100	50	50		
3	26	13	13		
Interval surgery–radiotherapy					Matched
≤4 weeks	76	38	38		
4–6 weeks	54	27	27		
≥6 weeks	26	13	13		

Exclusion criteria were as follows: (1) patients with esophageal cancer; (2) patients with adenocarcinoma, carcinoid, and small cell carcinoma; (3) postoperative pathology showing extrapulocapsular invasion and positive margin, and postoperative concurrent chemoradiotherapy; and (4) patients who did not complete postoperative radiotherapy.

### 2.2 Surgery

All patients in this cohort underwent bilateral cervical lymph node dissection. Laryngeal function was restored in 23 patients, while 133 patients underwent total pharyngectomy and total laryngectomy. Reconstructive methods were employed in 129 cases, including free skin graft transplantation in 7 cases, reconstruction with a pectoralis major myocutaneous flap in 22 cases, free jejunum transplantation in 82 cases, and gastric pull-up in 18 cases.

### 2.3 Postoperative radiotherapy

PORT was delivered at 2.0 Gy per day for five consecutive days using a 6 MV linear accelerator and was planned 2–4 weeks after surgery. All patients had no high-risk factors, such as ECE, and close (<5 mm) or positive surgical margins. Patients were treated with the whole-field intensity-modulated radiotherapy (IMRT) technique. The clinical target volume (CTV) included the former tumor bed plus bilateral cervical lymph node drainage areas (level II–V nodes and retropharyngeal nodes). The CTV was expanded by 0.5 cm to generate the planning target volume (PTV). In the LD-RT group, the target area was the surgical bed and cervical lymph node drainage areas, and the total dose was 50 Gy. Some patients with pathological N1–3 had no ECE, but CT scans showed bulky lymph nodes. After 25 planning sessions, a local dose of 10 Gy in 5 fractions of 2 Gy was delivered to the surgical bed of the large lymph node, resulting in a total dose of 60 Gy.

### 2.4 Propensity score matching

PSM was used to address confounding factors with the R package “MatchIt.” Matching variables included gender, age, subsite, tumor grade of differentiation, T-classification, N-classification, and Interval surgery–radiotherapy.

### 2.5 Statistical analysis

Statistical tests were performed using SPSS version 26.0. The Kaplan–Meier method was used to estimate OS, PFS, and LRFS. The log-rank test was used to compare survival status, and the chi-square test was used to compare baseline characteristics between groups. A *p*-value < 0.05 was considered statistically significant. Multivariate analyses were performed using Logistic regression modeling.

## 3 Results

### 3.1 LD-RT group

During the follow-up period, 9 out of 78 patients (11.5%) in the LD-RT group experienced locoregional recurrence, and 3 patients (3.8%) developed a second primary tumor, specifically 3 esophageal carcinomas. Additionally, 17 patients (21.7%) had distant metastases, with the most frequent location being the lung (9 patients, 53%).

### 3.2 HD-RT group

During the follow-up period, 48 patients survived, and 30 patients died in the HD-RT group. Among the 78 patients, 12 (15.3%) had locoregional recurrence, and 5 (6.4%) developed a second primary tumor. Moreover, 18 patients (23%) experienced distant metastases, with the lung being the most common location (13 patients, 72%).

### 3.3 Acute side effects of radiotherapy

Assessment of the extent of radiation-related toxicities was conducted according to the Radiation Therapy Oncology Group (RTOG) criteria (11). No grade 4 side effects were observed in either of the two groups. In the LD-RT group, the number of cases with grade 1, 2, and 3 acute mucositis was 36 (46.1%), 39 (50%), and 3 (3.9%), respectively. In the HD-RT group, the corresponding numbers were 22 (28.2%), 41 (52.6%), and 15 (19.2%), respectively. None of the patients in the entire group experienced grade 3 or higher acute radiodermatitis. In the LD-RT group, the number of cases with grade 1 and 2 acute radiodermatitis was 69 (88.5%) and 9 (11.5%), respectively, while in the HD-RT group, the numbers were 63 (80.7%) and 15 (19.3%), respectively, as shown in **Table 2**.

**Table 2:** Acute toxicity rates by treatment arm.

Grade	Low-dose Group(n=78)	High-dose Group(n=78)	*P*-value
Acute Mucositis			0.003
1	36	22	
2	39	41	
3	3	15	
Acute Radiodermatitis			0.183
1	69	63	
2	9	15	

### 3.4 Survival analysis

**Figure 1:**
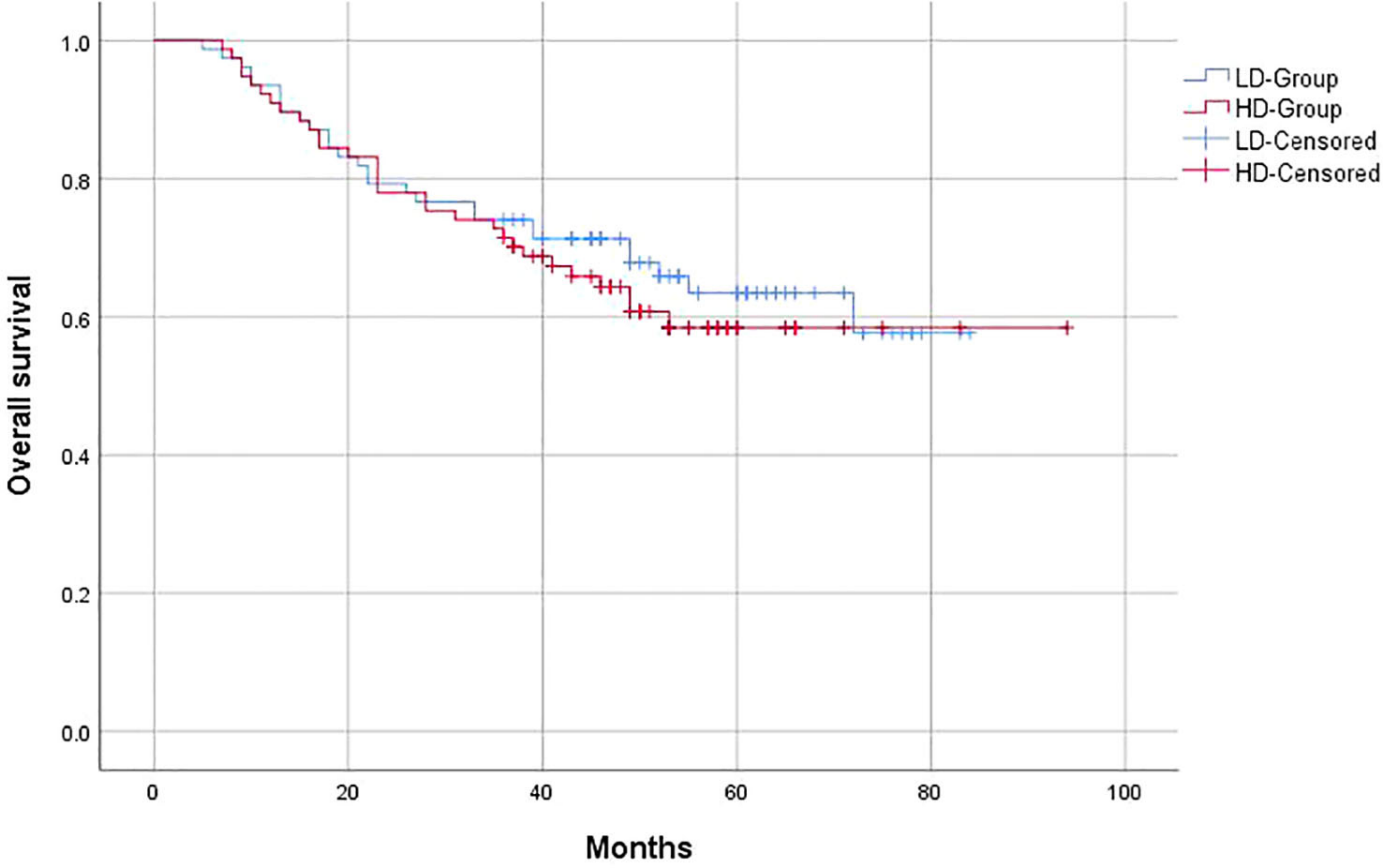
Kaplan–Meier curves of 5-year overall survival for hypopharyngeal cancer patients in LD-RT Group (blue line) and HD-Group (red line).

**Figure 2:**
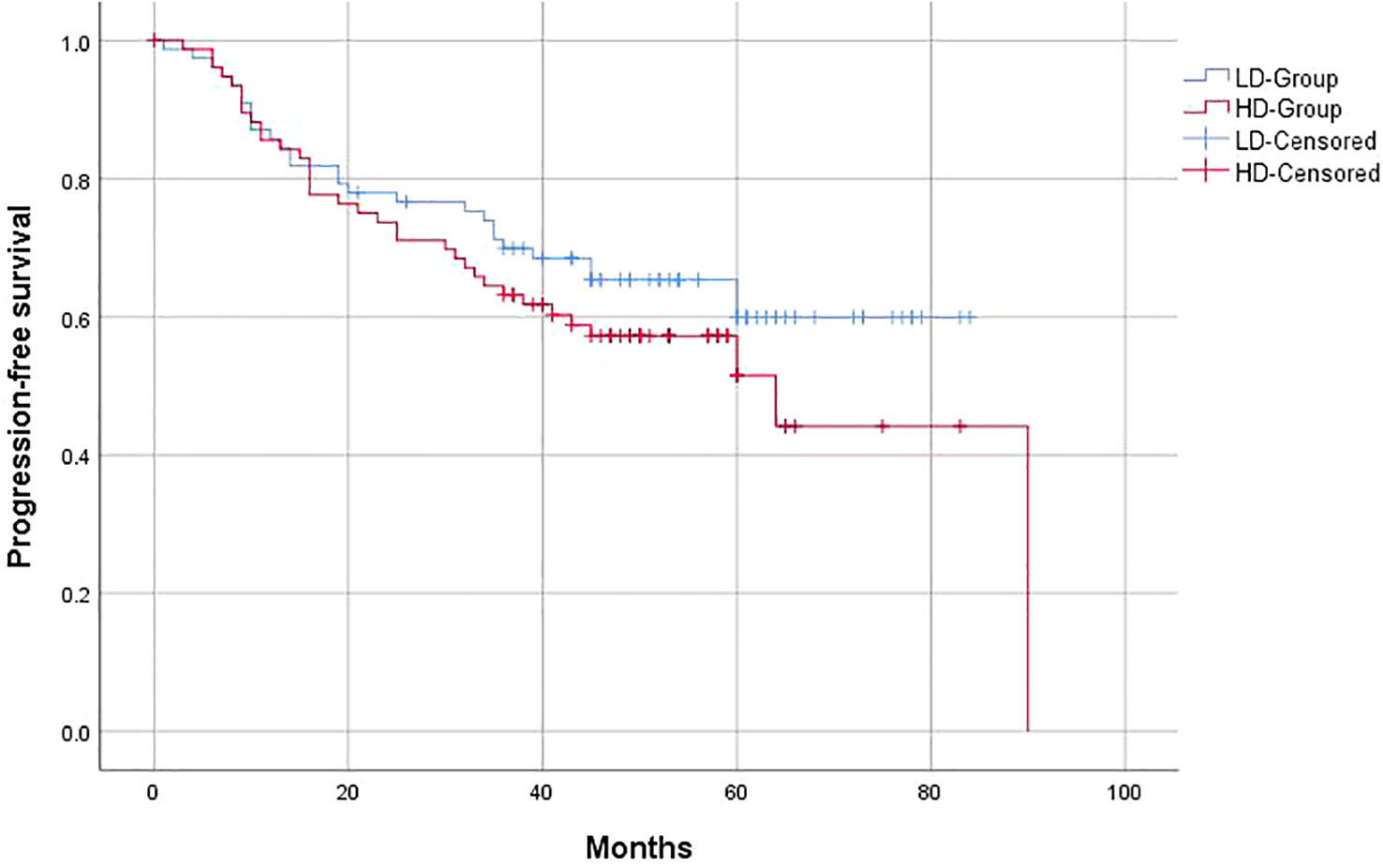
Kaplan–Meier of 5-year progression-free survival for hypopharyngeal cancer patients in LD-RT Group (blue line) and HD-Group (red line).

The 3-year OS of the entire group was 63%, and the PFS was 59.1%, with a locoregional control rate of 86.4%. In the LD-RT group, the 3-year OS and PFS were 64.9% and 63.6%, respectively. In the HD-RT group, the 3-year OS and PFS were 61% and 54.5%, respectively. Overall, there were no significant differences in OS (*p* = 0.547) and PFS (*p* = 0.25) in the matched-pair comparison between the LD-RT and HD-RT groups (**Figures 1**, **2**).

**Figure 3:**
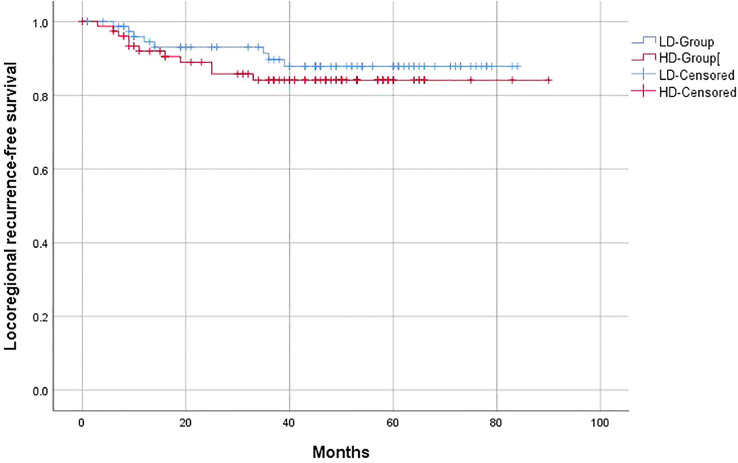
Kaplan–Meier curves of 5-year locoregional recurrence-free survival for hypopharyngeal cancer patients in LD-RT Group (blue line) and HD-Group (red line).

**Figure 4:**
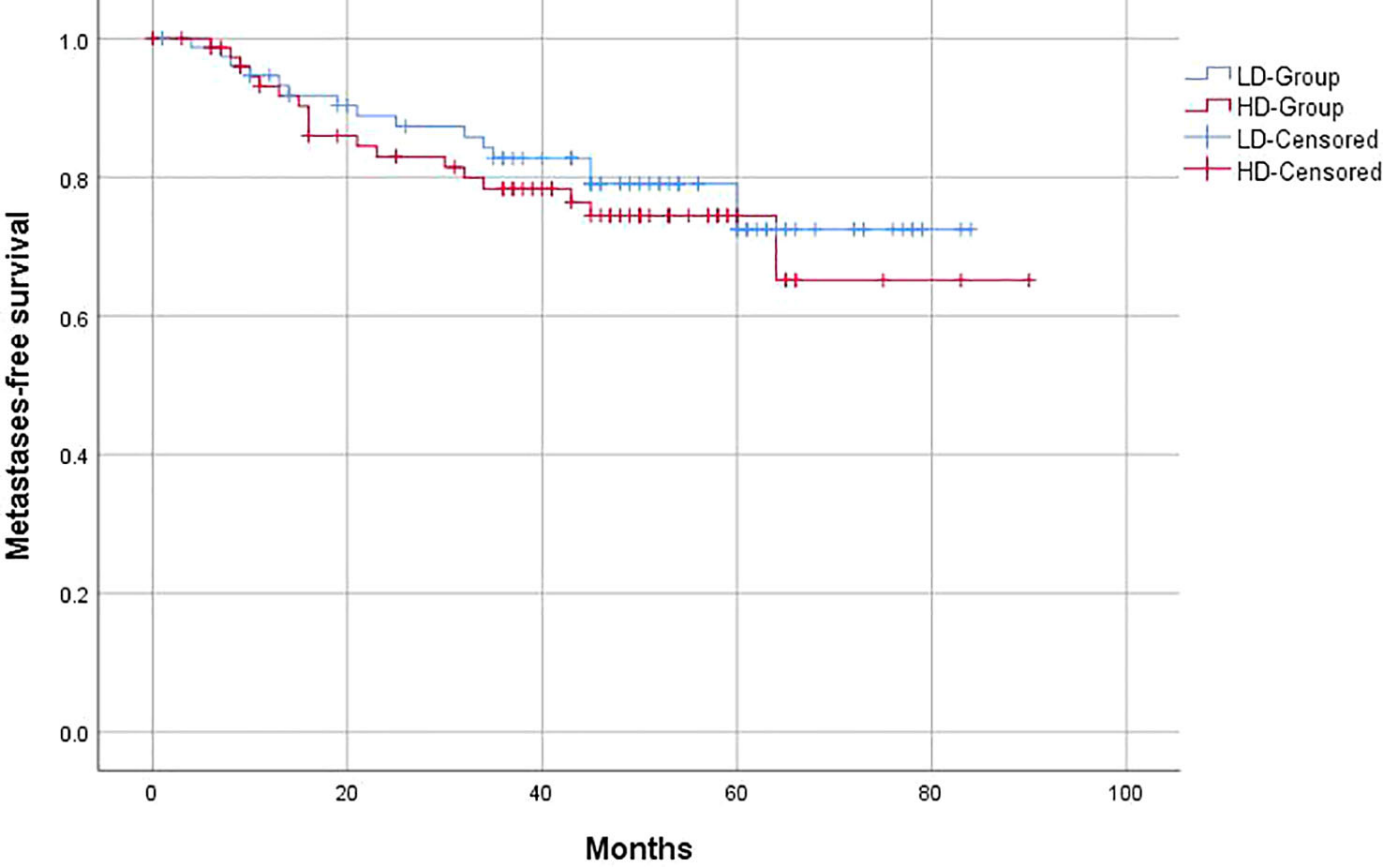
Kaplan–Meier curves of 5-year distant metastases-free survival for hypopharyngeal cancer patients in LD-RT Group (blue line) and HD-Group (red line).

**Figure 5:**
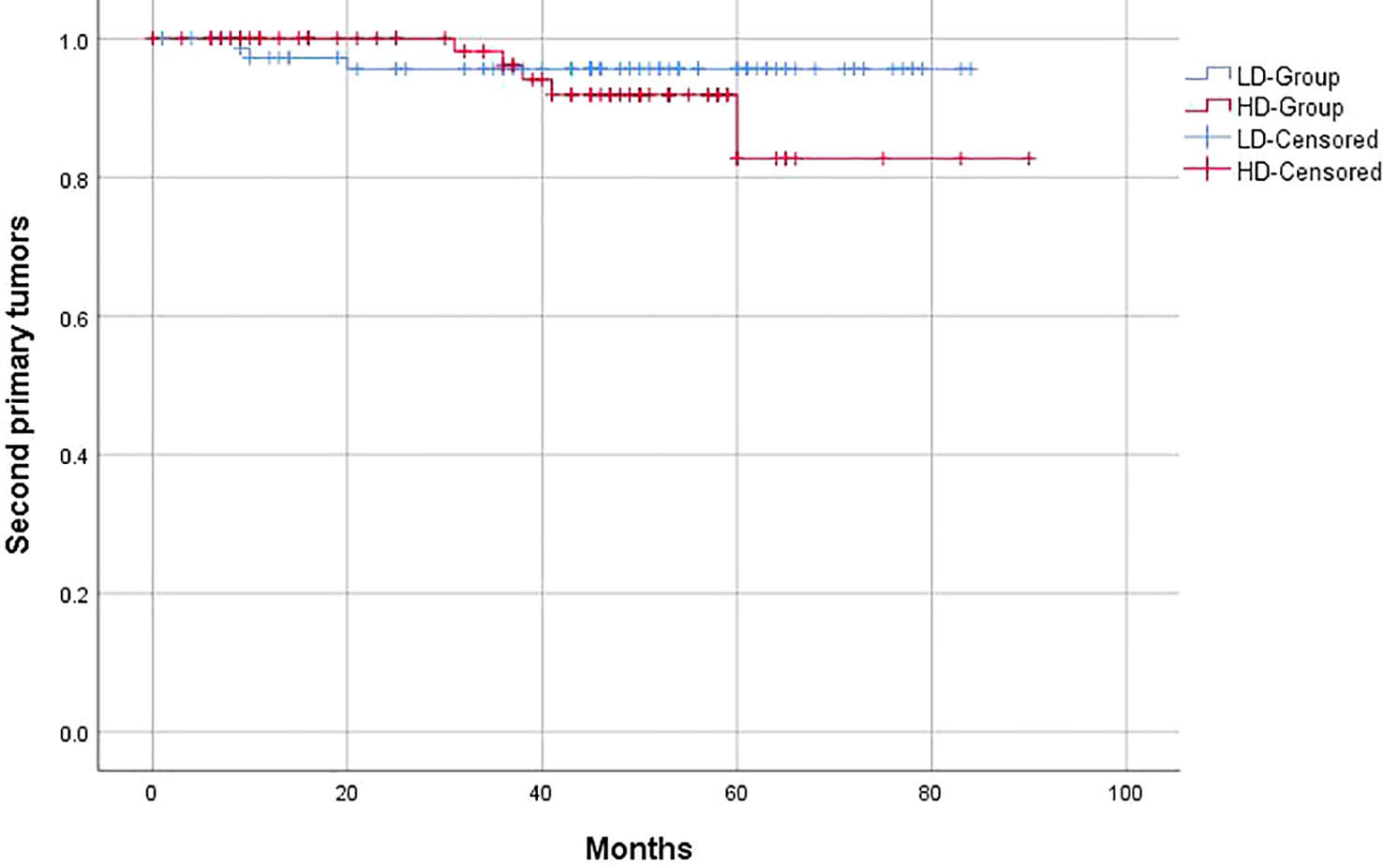
Kaplan–Meier curves of 5-year second primary tumor-free survival for hypopharyngeal cancer patients in LD-RT Group (blue line) and HD-Group (red line).

Similar to the OS and PFS results, there were no significant differences between the LD-RT group and HD-RT group for LRFS (89.6% vs. 85.7%; *p* = 0.439), distant metastases-free survival (DMFS) (79.2% vs. 76.6%; *p* = 0.506), and second primary tumor-free survival (SPTFS) (96.1% vs. 93.5%; *p* = 0.347) (**Figures 3**–**5**).

### 3.5 Prognostic analysis

Multivariate analyses revealed that pathological T and N classification were associated with 3-year OS, PFS, and LRFS, serving as independent prognostic factors (*p* < 0.05). However, gender, radiotherapy dose, and interval between surgery and radiotherapy were not associated with OS and PFS (**Table 3**). Moreover, compared with HD-RT, LD-RT was not associated with an increased risk for death and progression according to univariate analysis (**Table 4**).

**Table 3:** Multivariate Analysis of OS, PFS, and LRFS.

Factor	*OS*		PFS		LRFS	
	OR (*95%CI*)	*P value*	OR (*95%CI*)	*P value*	OR (*95%CI*)	*P value*
Age	0.967 (0.928-1.006)	0.097	0.958 (0.921-0.997)	0.035	0.977 (0.937-1.019)	0.286
Gender	0.208 (0.024-1.805)	0.154	0.272 (0.038-1.952)	0.195	0.857 (0.082-8.914)	0.897
T stage	2.682 (1.457-4.938)	0.002	2.585 (1.452-4.602)	0.001	3.242 (1.477-7.113)	0.003
N stage	3.336 (1.682-6.619)	0.001	2.809 (1.504-5.248)	0.001	3.478 (1.302-9.290)	0.013
Pathological differentiation degree	9.612 (3.115-29.661)	0.000	4.452 (1.694-11.700)	0.002	1.515(0.497-4.618)	0.465
Radiotherapy dose	1.214 (0.546-2.701)	0.634	1.870 (0.861-4.063)	0.114	1.102 (0.392-3.100)	0.853
Interval surgery–radiotherapy	0.571 (0.324-1.008)	0.053	0.739 (0.439-1.242)	0.253	0.857 (0.411-1.242)	0.681

**Table 4:** Odds ratios for event rate associated with LD-RT.

Univariable Analysis on Matching Variables	Value
Overall Survival	
Odds ratio	1.182
95% Confidence interval	0.614-2.275
*p*	0.617
**Progression-free survival**	
Odds ratio	1.458
95% Confidence interval	0.765-2.781
*p*	0.252
**Locoregional recurrence-free survival**	
Odds ratio	1.473
95% Confidence interval	0.544-3.797
*p*	0.464

## 4 Discussion

Surgery combined with radiotherapy is the main treatment approach for locally advanced hypopharyngeal carcinoma. However, guidelines provide varying recommendations regarding the dose of PORT, leading to differences in treatment protocols across medical centers. Early studies based on radiation biology suggested that doses of 45–50 Gy were sufficient to control microscopic disease, establishing postoperative radiotherapy as a standard treatment for hypopharyngeal cancer (12). In the 1990s, Peters et al. (2) demonstrated that regions of the neck with ECE should receive a boost to 63 Gy, while 57.6 Gy was sufficient for other risk factors. Similar results were reported by Ang et al. (13). However, Mohanti et al. (14) compared postoperative doses of ≤54 Gy with ≥60 Gy and found that higher doses of 60–63 Gy were needed for specific risk factors, including (1) age (≤50 years), (2) ≥4 positive lymph nodes, and (3) interval between surgery and radiotherapy >100 days. A prospective study by Chin et al. (15) administered 60 Gy irradiation to HPV-positive oropharyngeal cancer patients with positive margins and ECE, but it did not reduce LRFS. Makino et al. (16) applied lower radiotherapy doses than Western countries, with irradiation doses of 54 Gy (1.8 Gy/fraction) for the drainage area and 64.8 Gy (1.8 Gy/fraction) for high-risk patients, showing that low radiotherapy doses could achieve the desired therapeutic effect.

In the last two decades, there have been relatively few reports on the postoperative dose for HPSCC patients without high-risk factors. In our matched-pair analyses, we found no statistically significant differences in OS and PFS between the HD-RT and LD-RT groups, which is consistent with historical evidence (2, 12, 14). Additionally, the 3-year OS of the entire group was 55.8%, and the PFS was 56.5%. Avkshtol et al. (17) also observed similar results in their study, applying simultaneous integrated boost IMRT with irradiation doses of 60 Gy and 45 Gy to high-risk and low-risk areas, respectively, resulting in a 3-year OS of 62.3%. Kirke et al. (18) retrospectively evaluated 3,518 patients with advanced HPSCC and found a 3-year OS of 67.5% for surgery with adjuvant radiotherapy. Our study indicated that LD-RT was not associated with an increased risk of death and disease progression compared to HD-RT, according to univariate analysis. The purpose of increasing adjuvant radiation doses is to improve disease outcomes for high-risk HPSCC. However, our study found no statistically significant differences in the 3-year locoregional control rate between the LD-RT group (87%) and HD-RT group (81.8%). This observation is consistent with the preliminary study by Peters et al. (2), which showed that there was no dose response for tumor control in HPSCC patients without ECE with radiotherapy doses ≥ 57.6 Gy. These findings were further confirmed by a randomized controlled multicenter clinical study (13). A study by Stromberger et al. (19) also reported similar results, defining the surgical tumor bed and positive lymph node area as low-risk regions and treating them with 56 Gy. They concluded that the difference in locoregional control between high-risk and low-risk groups was not statistically significant, and most recurrence sites were within the area of high-dose irradiation. In 2017, the more than 20-year follow-up results of the Rosenthal et al. (20) trial further supported that increasing postoperative dose did not significantly improve local control. These results were confirmed by a retrospective study by Ashour et al. (21). The findings from these studies collectively suggested that increasing the dose of postoperative adjuvant radiation did not proportionally improve the local control rate. One possible explanation is that increasing the total time may offset any potential benefits of higher doses (22).

The NCCN recommendations on risk factor and adjuvant therapy were based on studies like RTOG 9501 and EORTC 22931 (3, 4). High-risk factors in RTOG-0234 included positive surgical margins and ≥2 positive nodes (23). In contrast, high-risk factors were defined as positive surgical margins and ECE, while intermediate-risk factors were defined as pathological T3–4 and N2–3 stages in the Stromberger et al. study (19). Ferris et al. (24) defined high-risk factors as surgical margins <1 mm, ECE, perineural invasion, and two or more positive nodes. A study by Nishimura et al. (25) indicated that positive surgical margins and ECE were the core high-risk factors. In the study by Avkshtol et al. (17), they defined high-risk cohort as ECE, positive margins, and/or ≥5 positive lymph nodes. Similarly, Makino et al. (16) defined positive surgical margins and ECE as high-risk factors while considering the tumor bed and cervical lymph node drainage area as low-risk areas. Despite the recommended risk factor classifications by NCCN guidelines, the definition of postoperative risk factors varied among different centers, particularly regarding whether T stage and N stage should be considered high-risk factors. As such, the appropriateness of defining T3–4 and N2–3 as high-risk factors requires further exploration.

Our results are consistent with historical evidence that increasing the dose of adjuvant radiation may lead to potential side effects and reduced quality of life (26, 27). In our study, the HD-RT group had a significantly higher incidence of acute mucositis compared to the LD-RT group.

In conclusion, our matched-pair analysis results indicate that postoperative low-dose radiotherapy does not show inferiority to high-dose radiation for patients with advanced hypopharyngeal cancer without positive surgical margins and ECE in terms of OS, PFS, locoregional control, and metastases-free survival.

## Data availability statement

The original contributions presented in the study are included in the article/supplementary material. Further inquiries can be directed to the corresponding author.

## Author contributions

Conceptualization, YW. Literature Search, HT. Data Collection, ZS, ZL. Writing –original draft, HT. Project administration, HT. Writing –review & editing HT. Supervision, YW. All authors read and approved the final version of the manuscript.
